# On traits matching and the modular organization of food web and occurrence networks

**DOI:** 10.1111/1365-2656.70234

**Published:** 2026-03-16

**Authors:** Dalmiro Borzone Mas, Pablo A. Scarabotti, Pablo A. Vaschetto, Patricio Alvarenga, Martin Vazquez, Matías Arim

**Affiliations:** ^1^ Biology Department Concordia University Montreal Quebec Canada; ^2^ Laboratorio de Ictiología Instituto Nacional de Limnología (UNL‐CONICET) Santa Fe Argentina; ^3^ Facultad de Humanidades y Ciencias, Departamento de Ciencias Naturales, Universidad Nacional del Litoral Ciudad Universitaria Santa Fe Argentina; ^4^ Departamento de Ecología y Gestión Ambiental, Centro Universitario Regional del Este Universidad de la República Maldonado Uruguay

**Keywords:** assembly processes, ecological network assembly, functional diversity, Neotropical floodplain, niche‐based mechanism—abundance‐based mechanism, Piscivorous fishes

## Abstract

1. Modularity and nestedness have been observed recurrently across different ecological networks, including food webs and occurrence networks. These patterns emerge from species‐level processes, where interactions and occurrences are determined by niche‐based and/or abundance‐based mechanisms. Abundance‐based processes promote nested networks with gradients in the number of links determined by species abundances. Niche‐based processes can promote modular structures due to differential spatial filters or trait matching in discontinuous gradients of predators and prey traits or nestedness due to gradients in the strength of environmental filters or trait limitation for consumption. Here, we explore the mechanisms driving species‐level interactions and the resulting network structure in both food webs and occurrence networks of piscivorous fishes from the Paraná River.
2. Our study focused on 16 species of piscivorous fish. We constructed occurrence networks (149 communities, 3010 observations) and food webs (113 prey species, 1271 trophic interactions). Using null models, we assessed modularity and nestedness in both types of networks, as well as the existence of significant deviations in the trait composition, functional diversity and community‐weighted mean among modules. Moreover, we assessed the relationship between species abundance and degree to identify the potential role of abundance‐based processes.
3. Occurrence networks and food webs exhibited a modular structure, with no evidence of nestedness. In both networks, niche‐based mechanisms played an important role. Each module showed a distinct representation of habitat types in occurrence networks and prey types in food webs. A significant relationship was also observed between predator abundance and the number of interactions or occurrences, suggesting that abundance‐based mechanisms also contribute to network organization.
4. Here, we are getting ahead in understanding the mechanisms driving ecological organization in piscivorous fishes from the Middle Paraná River. Although food webs and occurrence networks represent distinct dimensions, our results reveal a consistent pattern: both are shaped by a combination of abundance‐ and niche‐based processes. This convergence highlights shared principles of network assembly across contexts. By disentangling the contributions of these mechanisms, our findings advance ecological theory and highlight that protecting functional diversity and resource heterogeneity is essential for preserving the structure of ecological networks.

## Graphical abstract

What mechanisms shape ecological networks? In Paraná River piscivores, species traits and abundance scaling promote modular structures. Our findings reveal how communities self‐organize and highlight the conditions needed to sustain them.

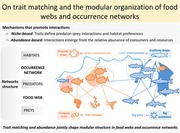

## 1 INTRODUCTION

The architecture of ecological networks determines the flow of individuals and energy across communities, thereby sustaining ecosystem functioning (Borzone Mas et al., 2025; Olesen et al., 2007; Thébault & Fontaine, 2010). Network structure emerges from mechanisms, such as the effects of species abundance and functional traits on interactions, the heterogeneity of resources and the filtering strength of consumer–resource interactions (Bartomeus et al., 2016; Delmas et al., 2019; Guimaraes Jr, 2020; Vázquez, 2005; Vázquez et al., 2005). Ecological interaction networks—including frugivory, pollination and food webs—consistently display two recurrent structural patterns: modularity and nestedness (Borthagaray et al., 2014; Olesen et al., 2007; Rezende et al., 2009). Nested networks are characterized by interactions of highly specialized species forming subsets of those observed in more generalist species (Mariani et al., 2019; Olesen et al., 2007). In contrast, modular networks consists of subgroups, that is modules, in which species interact more intensively among themselves and less frequently with species from other modules (Newman, 2006; Rezende et al., 2009). Although modularity and nestedness are often described as antagonistic patterns (Pinheiro et al., 2022; Trøjelsgaard & Olesen, 2013), they frequently coexist within the same system, forming composite topologies (Felix et al., 2022; Lewinsohn et al., 2006). For instance, networks may exhibit modular organization at a broad scale, while species within modules follow a nested pattern (Lewinsohn et al., 2006; Pinheiro et al., 2022; Felix et al., 2022). These network structures determine key ecosystem properties related to stability and functioning (Rodriguez et al., 2022; Nie et al., 2023; Mintrone et al., 2025). For example, spatial modularity promotes segregation between modules, reducing the spread of parasites and diseases in bat roosts and thereby enhancing stability (Fortuna et al., 2009). Similarly, modularity in food webs, through complementary effects, has been shown to promote biomass in fish communities (Borzone Mas et al., 2025).

**FIGURE 1:**
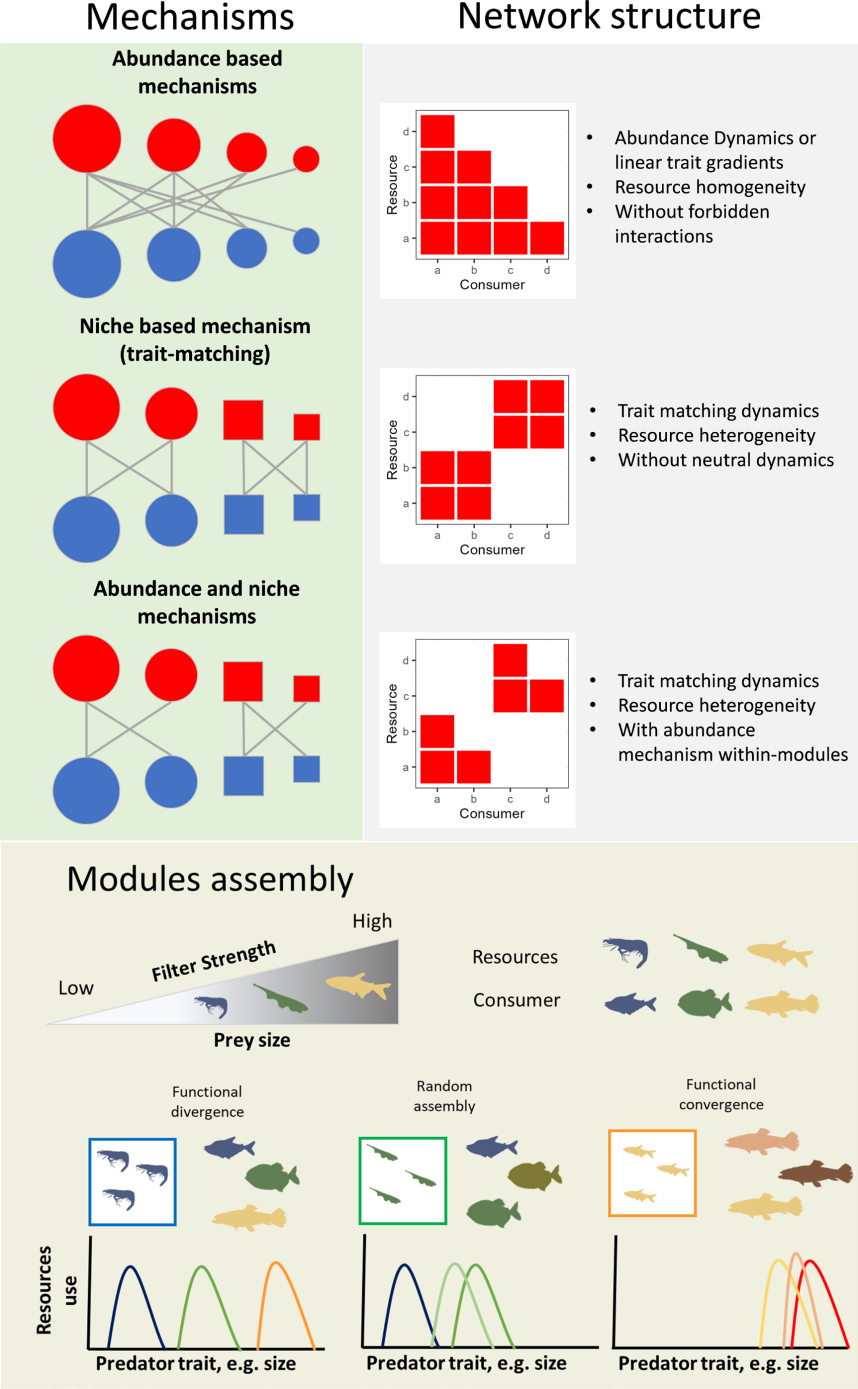
Hypothetical relationships between abundance and trait‐based mechanisms, network structure and module assembly. The first column illustrates interactions between consumers (red) and resources (blue), where figure size represents abundance and figure shape (circles vs. squares) indicates morphological diversity. The second column depicts the resulting network structures under different resource mechanisms and heterogeneity. The second row (module assembly) illustrates how modules form according to resource‐based access filters, exemplified here with prey body size and predator body size. The figures inside the square represent the prey that make up the module, while on the right are the predators that capture them. Depending on the strength of these filters, different assembly outcomes are expected: Resources with weak filters tend to produce modules with high functional divergence, whereas strong filters promote modules characterized by functional convergence. In this context, small prey can be consumed by predators of a wide range of body sizes, leading to greater functional divergence. Conversely, as filter strength increases (e.g. larger prey), only large predators can access these resources, resulting in stronger functional convergence within modules.

The fundamental mechanisms structuring ecological networks are abundance‐ and trait‐based processes, both of which promote interactions between consumers and resources (Dehling et al., 2025; Marjakangas et al., 2022; Ortiz et al., 2023; Vázquez, 2005; Vizentin‐Bugoni et al., 2014; Figure 1). These mechanisms operate differently depending on whether consumer traits influence the efficiency of resource use (Vázquez, 2005). Abundance‐based mechanisms highlight that interactions are determined by the probability of encounters between consumers and resources (Vázquez, 2005; Vázquez et al., 2005, 2009). This probability largely depends on the relative abundance of both groups within the community (Vázquez et al., 2009). In contrast, trait‐based mechanisms involve consumer and resource traits that directly influence consumption, such that interactions occur only when there is a functional ‘match’ between consumer traits and resource characteristics (Izquierdo‐Palma et al., 2021; Ortiz et al., 2023; Vázquez et al., 2009). For example, freshwater fish with an orbicular morphology tend to prefer habitats with little current, while more fusiform fishes tend to select habitats with stronger flow (Borzone Mas et al., 2022; Breda et al., 2005; Webb, 1984). In this way, habitat characteristics tend to be positively related to specific fish morphologies (i.e. consumers), thereby promoting interactions and influence the spatial use. Despite this strong theoretical foundation, our understanding of how these processes shape the structural complexity of ecological networks remains incomplete (Eskuche‐Keith et al., 2023; Pinheiro et al., 2019).

In addition to abundance‐ and trait‐matching mechanisms, resource heterogeneity and the filtering strength in consumer–resource associations also shape module assembly (Delmas et al., 2019; Mouillot et al., 2007; Pinheiro et al., 2019). Resource heterogeneity can be understood as the occurrence of distinct habitat types in space (e.g. lotic vs. lentic environments in freshwaters) or different prey categories in trophic interactions (e.g. invertebrates vs. fish, Borzone Mas et al., 2022; Sutton et al., 2021). Filtering strength, in turn, refers to the degree of ‘fit’ between consumer traits and the minimum requirements to exploit a given resource (Legras et al., 2019; Mouillot et al., 2007). These mechanisms interact to generate distinct modular architectures: abundance‐based processes often yield modules with random trait distributions (or even non‐modules promotion), whereas trait‐based processes imply a consistent association between traits and resource use (Eskuche‐Keith et al., 2023; Vázquez et al., 2009). Resource heterogeneity further modulates the relative importance of these mechanisms. Homogeneous resources promote abundance‐driven assembly, where generalist consumers exploit a wide variety of resources, resulting in modules with weak or random trait signals (Pinheiro et al., 2019). In contrast, heterogeneous resources constrain generalism and favour trait‐based filtering so that consumers require specific attributes to exploit particular resources, leading to modules structured around distinctive traits (Pinheiro et al., 2016; Pinheiro et al., 2019). For example, in mutualistic networks, convergence between hummingbird bill morphology and corolla curvature fosters the formation of modules, while in marine food webs, traits such as body size, mobility and trophic level drive modular organization (Eskuche‐Keith et al., 2023; Izquierdo‐Palma et al., 2021; Rezende et al., 2009). The strength of resource filters also plays a central role: when resource exploitation strongly depends on trait in consumer–resource associations, consumers with similar attributes cluster into functionally homogeneous modules (e.g. environmental filtering, Mouillot et al., 2007, Legras et al., 2019). Conversely, when barriers to resource acquisition are weak (Sutton et al., 2021), competition among consumers (e.g. through spatial or trophic segregation) becomes the dominant force, promoting functional divergence within modules (Mouillot et al., 2007). Thus, while trait matching tends to generate functional convergence across modules, competition drives within‐module differentiation (HilleRisLambers et al., 2012; Mouillot et al., 2007).

In this way, it is possible to infer how abundance, and trait matching, resource heterogeneity and the filtering strength of consumer–resource associations shape network structure and module assembly. When abundance‐based mechanisms dominate in systems with homogeneous resources, nested networks are expected, with consumer degree directly associated with abundance (Ortiz et al., 2023; Pinheiro et al., 2019). Similarly, when consumer traits are linearly or unimodally related to resource consumption or space use efficiency, nestedness is promoted through gradients in consumption efficiency (Olesen et al., 2007; Omidipour et al., 2021; Ortiz & Arim, 2016). Alternatively, when trait‐based mechanisms prevail in heterogeneous systems, modular networks are expected, with modules oriented towards specific resources and consumers characterized by particular traits (Olesen et al., 2007; Pinheiro et al., 2019). In cases where trait‐based mechanisms promote modules but abundance‐based mechanisms act within them, composite topologies may emerge, with a modular network organization and nestedness within modules (Felix et al., 2022). Finally, the filtering strength of consumer–resource associations determines modular assembly: when resources impose strong filters, only consumers with specific traits are represented in each module, leading to low functional diversity as functionally similar consumers exploit the same resources (Legras et al., 2019; Mouillot et al., 2007). Conversely, when trait barriers are weak, competition promotes consumer differentiation, increasing functional diversity within the module (Figure 1, Mouillot et al., 2007).

In this work, we assess the main predictions of the previously stated hypotheses regarding the mechanisms that generate structural patterns in occurrence networks and food webs of piscivorous fish species in the Middle Paraná River. Piscivorous predators exhibit remarkable differences in traits, including highly divergent body sizes, teeth and body morphologies, and wide variation in resource use and tolerance to environmental conditions (Borzone Mas et al., 2022). Given their ability to regulate prey dynamics, enhance connectivity across network compartments and facilitate nutrient recycling, predators are essential components of ecosystems (Borzone Mas et al., 2022; Hammerschlag et al., 2019). Thus, understanding the mechanisms underlying community organization becomes a priority task. While occurrence networks capture habitat‐driven co‐distribution and constraints associated with environmental heterogeneity, food webs reflect direct consumer–resource interactions that mediate energy flow and species coexistence. Since they constitute key and complementary aspects of the ecosystem, the simultaneous analysis of the mechanisms underlying the organization of occurrence networks and food webs provides a more integrative understanding of how biodiversity is structured and maintained (Borzone Mas et al., 2022). As expected, fish communities in the Middle Paraná show a persistent modular organization in both food webs and occurrence networks (Borzone Mas et al., 2022). In this context, the combination of high resource heterogeneity and predator diversity makes the system an ideal natural laboratory to investigate the processes driving module formation and the interplay between trophic and spatial dimensions of community structure.

## 2 METHODOLOGY

### 2.1 Field sampling

The Paraná River is among the world's top 10 largest rivers, traversing a region characterized by a pronounced climatic seasonality and displaying considerable hydrological variability and geomorphological complexity (Scarabotti et al., 2017). Seasonal floods consist of water elevations of approximately 2–3 m above the annual mean level standing on average between 2 and 6 months. Similar to other large rivers in temperate and subtropical regions, the timing and duration of floods, along with their alignment with warm temperature periods, strongly influence fish community composition and trophic ecology (Scarabotti et al., 2011, 2017). Fish were sampled across 28 water bodies encompassing four main habitat types—main rivers (MR), secondary channels (SC), connected lakes (CL) and isolated lakes (IL)—which differ in flow, size and hydrological connectivity. Main rivers (MR) were wide lotic environments (330–1100 m), secondary channels (SC) were narrower (28–137 m) and meandered through floodplain islands, connected lakes (CL) were lentic systems permanently linked to channels (3.4–240 ha), and isolated lakes (IL) were temporarily disconnected from channels (4.1–40.1 ha). Sampling was conducted between 2013 and 2017, covering different hydroclimatic conditions defined by the combination of hydrometric level (high vs. low water) and thermal season (warm vs. cold). In total, 149 samples (hereafter referred to as communities) were collected, each representing a unique combination of site and sampling date. A detailed list of sampling sites, habitat types and sampling dates is provided in Material S1.

Fish were collected using standardized gillnets and beach seine nets (see Borzone Mas et al., 2022 for details). Each individual was identified, measured and weighed. Twelve ecomorphological measurements were taken on one adult individual of each piscivorous species (Table S1 and Material S2). Morphological traits were rather stable within each species and intraspecific variation was very low in comparison with differences between species (personal observation). These variables were selected following the methodology of Villéger et al. (2011), Winemiller (1991) and Claverie and Wainwright (2014), and were related to mobility, speed, acceleration and predatory behaviour. Fish stomachs were preserved in 10% formalin and examined in the laboratory under a binocular microscope. Fish prey was identified at the species level, while invertebrates were identified at the order level. Preys were classified into the following groups: Characiformes, Siluriformes, Gymnotiformes, Perciformes, other fish orders, invertebrates, fish remains and plant remains. As large numbers of juveniles of *Salminus brasiliensis* were collected in the area and this species exhibits strong ontogenetic differences in habitat use and feeding (Rossi et al., 2007), we divided individuals of this species into juvenile (<1000 g) and adult for both food webs and spatial networks. Fish sampling was approved by the Ministry of Environment of the province of Santa Fe (Resolution 036/18) and followed ethical recommendations of the National Council of Scientific and Technical Research of Argentina (CONICET, 2005).

### 2.2 Network structure

Networks were built for occurrence and trophic interactions. The matrix of 16 species and 149 spatiotemporal communities (28 sites sampled at different frequencies, see Material S1) was used to build a weighted occurrence network, in which species abundance was considered. In the occurrence matrix rows corresponds to sites at specific times, the columns to the predator species, and the value in the cells represents the abundance of each predator at the site and at a specific time. This implies that spatial modules emerge when predators coexist in both space and time. With the trophic interactions observed in each of the 149 samples, we build a unique food web. The food web was built from the occurrence of prey in the stomachs of individuals of piscivorous species captured throughout the duration of the study in all 149 communities. For both fish and plants, remains were identified based on colour, scales or other distinguishable organoleptic properties. In the food web matrix, rows correspond to prey types (113 prey items), the columns to predator species and cell values to the frequency of occurrence of a given prey species on the stomach of a predator species.

We calculated the modularity index (Qw) and determined module membership for both the occurrence network and the food web using the Simulated Annealing algorithm (Guimera & Amaral, 2005). Given the different nature of these interaction types, we used the RNetcarto (Doulcier & Stouffer, 2015) package for weighted and directed unipartite networks (food webs) and Beckett's (2016) method implemented in the bipartite package (Dormann et al., 2008) for weighted bipartite networks (occurrence networks). Because modular memberships are obtained through an optimization process that can yield different results across runs, we repeated the procedure 100 times for each network and retained the configuration with the highest modularity value as the final network structure.

In occurrence networks, analysis of modularity identifies groups of consumers that tend to use a common set of sites more frequently than others (modules), whereas in food webs, modules represent groups of consumers that tend to exploit a shared subset of prey. Because modularity (Qw) values are network‐specific and highly sensitive to intrinsic properties, such as network size and connectance (Beckett, 2016), we report normalized modularity values (Dormann et al., 2008; see the Trait Matching section). We also quantified the degree of nestedness in both networks using the WNODA metric (Weighted Nestedness based on Overlap and Decreasing Abundance; Almeida‐Neto et al., 2008), which ranges from 0 (perfectly non‐nested) to 100 (perfectly nested). WNODA values were calculated for both the complete network and within each module (Felix et al., 2022; Pinheiro et al., 2022). The statistical significance of modularity and nestedness for both the occurrence networks and food webs was evaluated using the null model proposed by Vázquez et al. (2007), which strictly preserves matrix connectance while maintaining probabilistically species degree distribution. This model allows testing whether networks are more modular or nested than expected given stochastic assembly constraints, such as connectance and degree distribution (Felix et al., 2022; Vázquez et al., 2007). Additionally, to assess intramodular nestedness, we followed the approach of Felix et al. (2022), which complements the Vázquez null model by keeping species membership within each module constant. Since different modules can exhibit different levels of internal nestedness, we compared nestedness values for each module (those with more than one predator species) and at the network level with the respective simulated values. To analyse the effect of abundance‐based mechanisms, we related the abundance of each predator with their degree in each network (number of communities used in the spatial network and number of prey items in the food web).

### 2.3 Assembly rules in the formation of network modules

**FIGURE 2:**
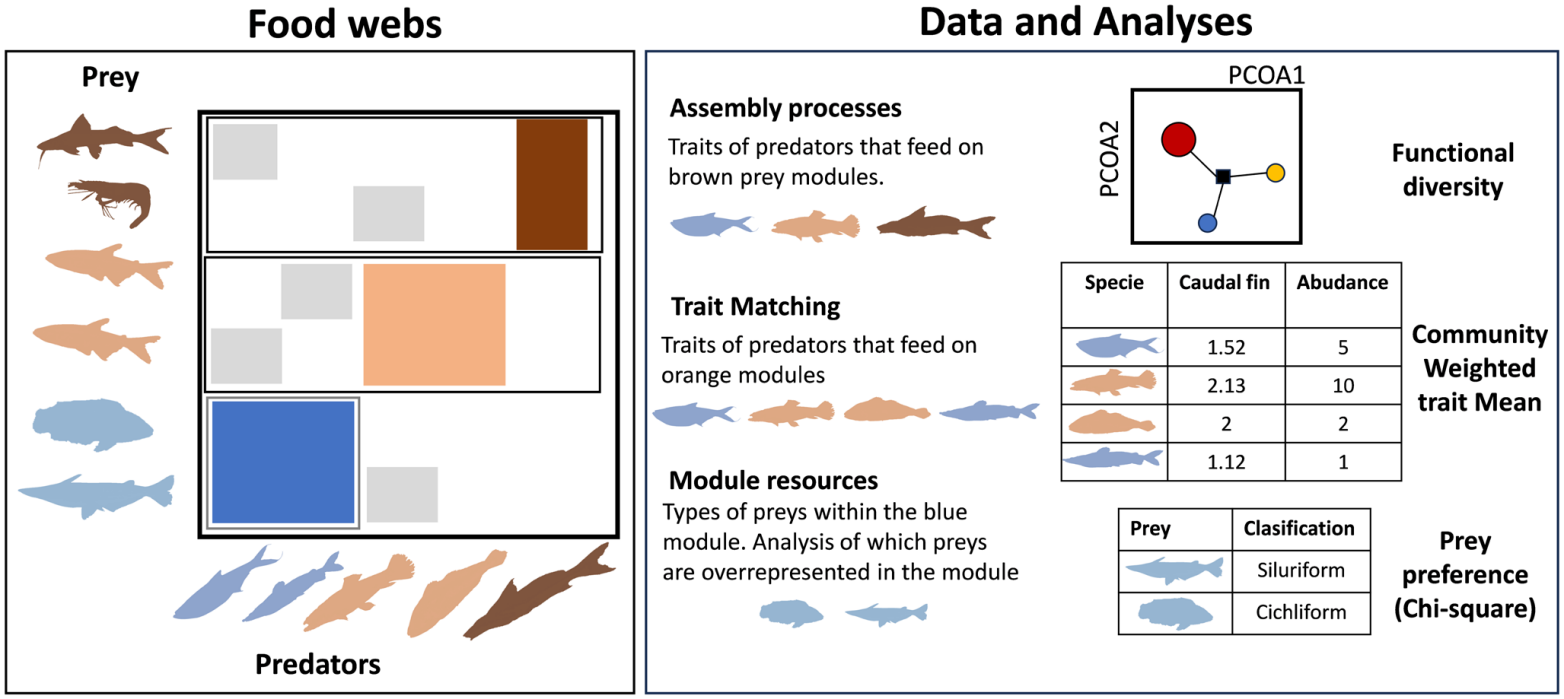
Methodological summary of the analyses performed at intramodular level. The food web represents a network where the rows are prey and the columns are predators. Organisms of the same colour represent the same module, intramodular interactions are coloured with the colour of the module while intermodular interactions (outside the module) are grey. The ecological concepts column highlights the data considered for each analysis. The analyses for the occurrence network were performed in the same way.

To explore the assembly processes within modules, we quantified the functional diversity of consumers exploiting the resources grouped into each module. We used a functional trait matrix comprising 12 variables related to resource use (e.g. tooth type, mouth position, gape size; see Table S1), following Villéger et al. (2011) and Winemiller (1991). Functional diversity was estimated as Functional Dispersion (FDis) with the R package FD (Laliberté & Legendre, 2010). FDis represents the mean distance of consumer species to the centroid in a multidimensional trait space, with distances weighted by consumer relative abundances. Consequently, modules with low FDis values indicate strong convergence in consumer traits, whereas high FDis values reflect trait divergence (Laliberté & Legendre, 2010). To compute FDis, we first identified all resources used by the species of a module and then evaluated how all consumers in the network (not only those belonging to a that module, see Figure 2) exploited the resources of this module and calculated FDis for these consumers. To test whether the observed functional diversity differed from random expectations, we generated 2000 null modules for each empirical module using Monte Carlo–based randomizations. Null modules were built by resampling interactions from the complete network while maintaining the number of interactions per module. This design accounts for the possibility that resources within a module may present weak barriers, allowing the access of consumers from other modules to them and thereby increasing functional diversity. Importantly, this approach enabled us to calculate FDis for all modules, including monospecific ones, and ensured that values reflected the functional diversity of the entire consumer assemblage.

### 2.4 Trait matching

To evaluate the role of trait‐matching mechanisms in network assembly, we compared predator traits with the resources (sites or prey) associated with each module. In occurrence networks, trait matching was assessed between predator traits and the set of sites within each module, while in food webs, it was evaluated between predator traits and the set of prey types defining each trophic module. For categorical traits (e.g. tooth shape, pigmentation and oral gape position), we calculated the frequency of each trait category within modules and compared it to the expected frequency based on the entire network composition. Expected (proportion of traits in whole network) and observed frequencies were arranged in contingency tables, and significance was assessed using chi‐square tests. For quantitative traits (e.g. body size, caudal fin aspect ratio), we calculated the community‐weighted mean (CWM) of predator traits within each module, weighting by species abundance. Following the same null‐model approach used for functional diversity, we evaluated CWM by generating 2000 null modules per empirical module. For prey and predator body size, we used individual‐level measurements, whereas all other traits were considered at the species level. This procedure generates random networks that preserve module size but randomize the identity of interacting species (consumer–prey in food webs, consumer–site in occurrence networks). This approach allows the detection of non‐random associations between predator traits and the types of resources exploited (sites or prey), providing evidence for trait matching in both networks.

For nestedness metrics (WNODA and SM‐WNODA), modularity (Qw), *FDis* and CWM, we evaluated deviations from null expectations using standardized z‐scores calculated as follows:

$$z=\frac{X_{\mathrm{obs}}-\bar{X}}{\mathrm{Sd}\left(X\right)}$$

where $X_{\mathrm{obs}}$ refers to the observed metric, $\bar{X}$ the mean value of simulated metrics $\mathrm{Sd}\left(X\right)$ the standard deviation of simulated value. The standardized *Z* values greater than |*z*| > 2 were considered as significant (see also Table 1). In addition, deviations from random expectation in the representation of prey and habitats within modules was assessed with contingency tables and then evaluated by chi‐squared tests (Table 1). In the occurrence networks, each site was classified according to habitat type as isolated lake, connected lake, secondary channel and major river. The expected proportion for the contingency test was the average frequency of occurrences among habitat types along all the species. For the food web, the expected proportion corresponded to the proportion of each prey category in the whole piscivorous assemblage. Finally, all statistical analyses were performed using R version 4.2.0 (R Core Team, 2022). This work used computational resources from the Pirayu cluster, acquired with funds from the Santa Fe Agency for Science, Technology and Innovation (ASACTEI), Government of the Province of Santa Fe (through project AC‐00010‐18, resolution 117/14).

**TABLE 1:** Metrics analysed, level of each metrics, null models and the implications of observed patterns.

Metrics	Level of ecological organization	Null model	Obs > 2 z	Obs < −2 z
WNODA	Whole network	Fixed connectance and probabilistic degree distribution	Nested network	Anti‐nested network
WNODA‐SM	Intramodular process	Fixed connectance, degree distribution and modular membership restriction	Nested intramodular distribution	Anti‐nested intramodular distribution
Modularity	Whole network	Fixed connectance and probabilistic degree distribution	Modularity pattern	Anti‐modularity pattern
Fdis	Intramodular process	Monte Carlo Randomizations	Limiting similarity	Niche filtering
CWM	Intramodular process	Monte Carlo Randomizations	Trait matching	Trait barrier

## 3 RESULTS

**FIGURE 3:**
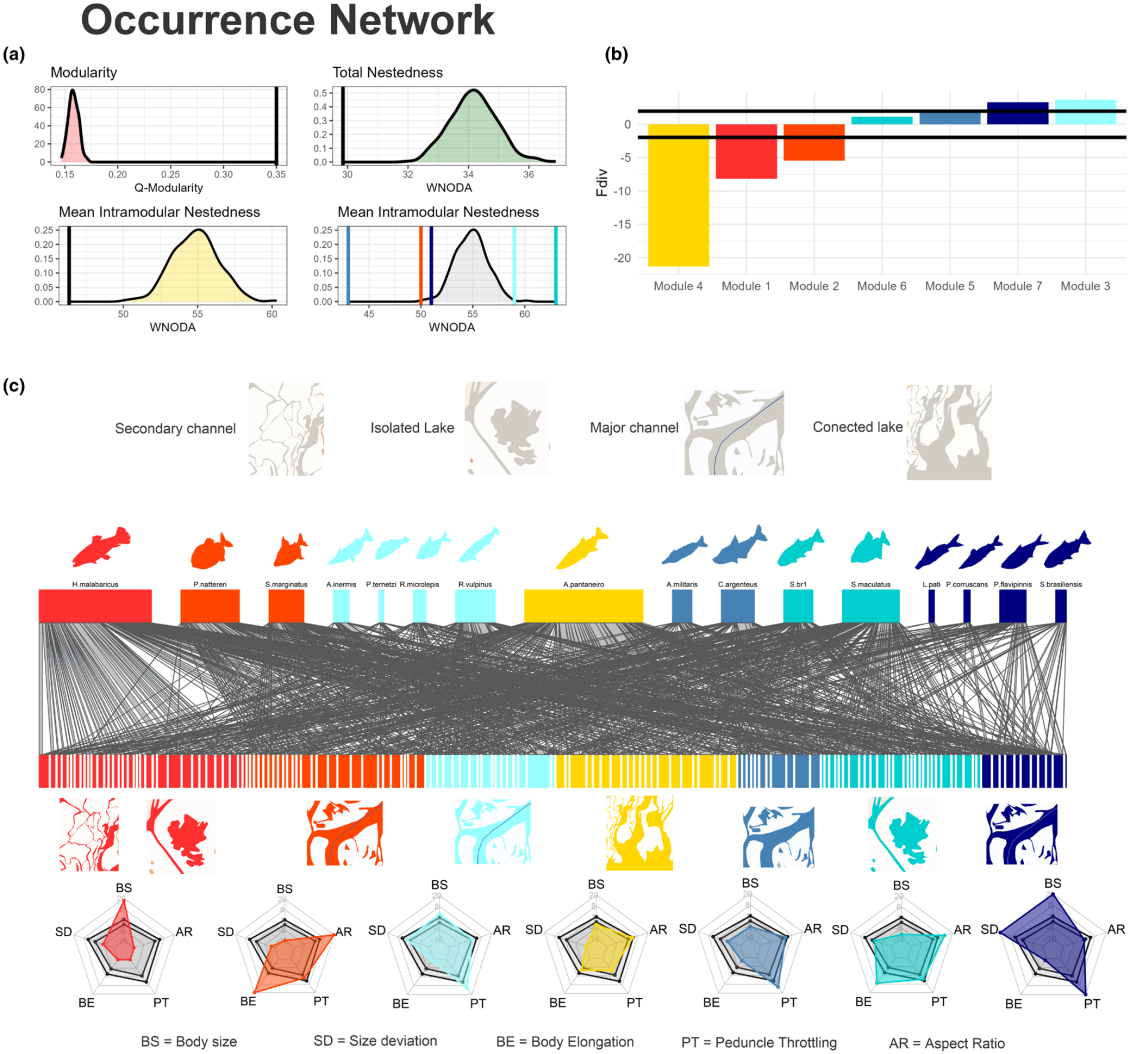
Occurrence network of predators in the Middle Paraná River. (a) Null‐model frequencies for modularity (red), total nestedness (green), mean within‐module nestedness (yellow) and for each module (grey). Black vertical lines represent observed values for each variable. For intramodular nestedness, the colour representation for each module can be read later in the legend. (b) Functional diversity values for each module, shown relative to selected habitat types. Black horizontal lines mark the bounds of null distributions (upper line = 2, lower line = −2), with bars representing observed values. (c) Different modules are represented by distinct colours, with fish illustrations in matching colours corresponding to the same module. Below the bipartite network, icons represent the habitat types most frequently used by species within each module. From left to right, modules correspond to: Module 1, generalist predators of lagoons; Module 2, scrapers in the main channel; Module 3, predators of medium and small river channels; Module 4, predators of connected lagoons; Module 5, small predators of main channel; Module 6, predators of isolated lagoons; and Module 7, large predators of main channel. At the bottom, radar charts show the community‐weighted mean (CWM) values for five traits (TD = predator body size, SD = predator standard deviation, EC = body elongation, EP = caudal peduncle compression, AR = caudal fin aspect ratio). The inner pentagon (light grey) marks the lower significance threshold (*z* = −2), and the outer pentagon marks the upper significance threshold (*z* = 2).

**FIGURE 4:**
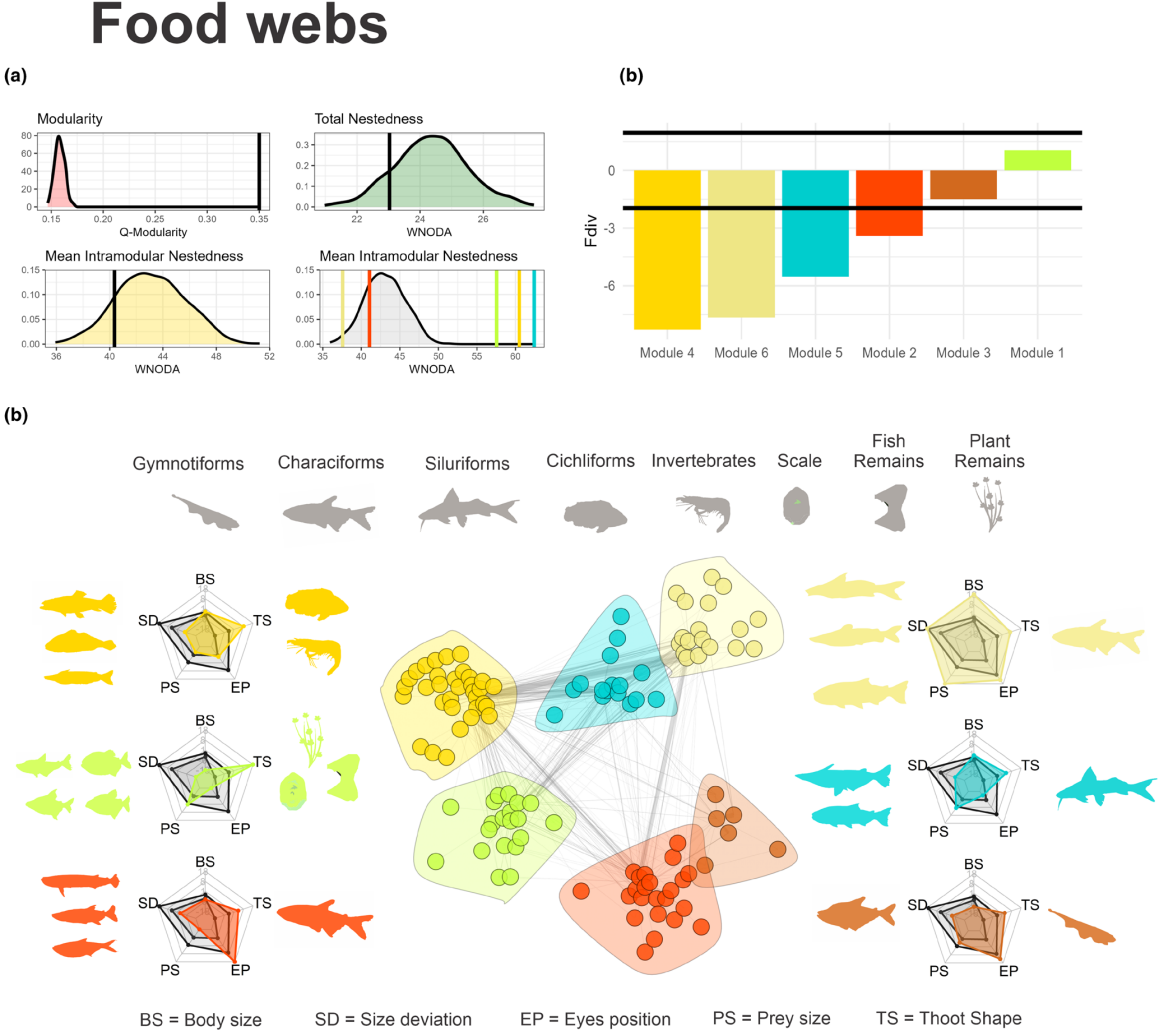
Food web of predators in the Middle Paraná River. (a) Null‐model curves for total modularity (red), nestedness (green) and mean within‐module nestedness (yellow) and for each module (grey). Black vertical lines mark the observed values for each variable. (b) Functional diversity values for each module. For intramodular nestedness, the colour representation for each module can be read later in the legend. Black horizontal lines represent the bounds of null distributions (upper line = 2, lower line = −2). (c) Unipartite network showing predator–prey interactions among 16 piscivorous species and their prey. The width of the lines determines the interactions between predators and prey. The colour of the nodes represents module membership. The pentagon charts show the community‐weighted mean (CWM) values for five traits (TD = predator body size, SD = predator size deviation, FD = tooth morphology, EP = eye position, PS = prey size). The inner pentagon (light grey) marks the lower significance threshold (z = −2), and the outer pentagon the upper significance threshold (*z* = 2) based on null values for these traits. The values for TS represent the chi‐squared test statistic. To the left of the pentagons are the predators' members of each module, while to the right are the significant prey of each module. The names and colours of the modules on the left, from top to bottom, are as follows: Module 4, generalist piscivores (gold); Module 1, scrapers (green); Module 2, predators of Characids (red) and the names of the modules on the right, from top to bottom, are as follows: Module 6, predators of open‐water/river channel detritivores (khaki). Module 5, predators of Siluriformes (cyan) and Module 3, predators of Gymnotids (brown).

The occurrence network had 16 species observed in 149 communities (site by date), totalizing 3010 interactions (species by community). The food web included 1271 interactions from 3010 observed stomachs distributed among 113 prey species. Data for each species, such as abundance and degree in occurrence networks and food webs, can be seen in Table S2. At the network level, both the occurrence network and the food web exhibited a modular structure (*ZQ* = 23.32 for the occurrence network and *ZQ* = 40.13 for the food web) with seven modules on the occurrence network and six on the food web (Figures 3 and 4). The nestedness of the occurrence network was lower than expected by chance (*WNODA* matrix = −3.26 z, *WNODA‐SM* = −3.09 z, Figure 3a), whereas the nestedness of the food web did not differ from chance (*WNODA* matrix = −1.13 z, *WNODA‐SM* = −1.05 z, Figure 4a). The species included in each module and the resources exploited by them can be observed in Figure 3 for the occurrence network, and in Figure 4 for the food web. The identity of the members of each module for the occurrence network and the food web can be seen in Tables S3 and S4.

**FIGURE 5:**
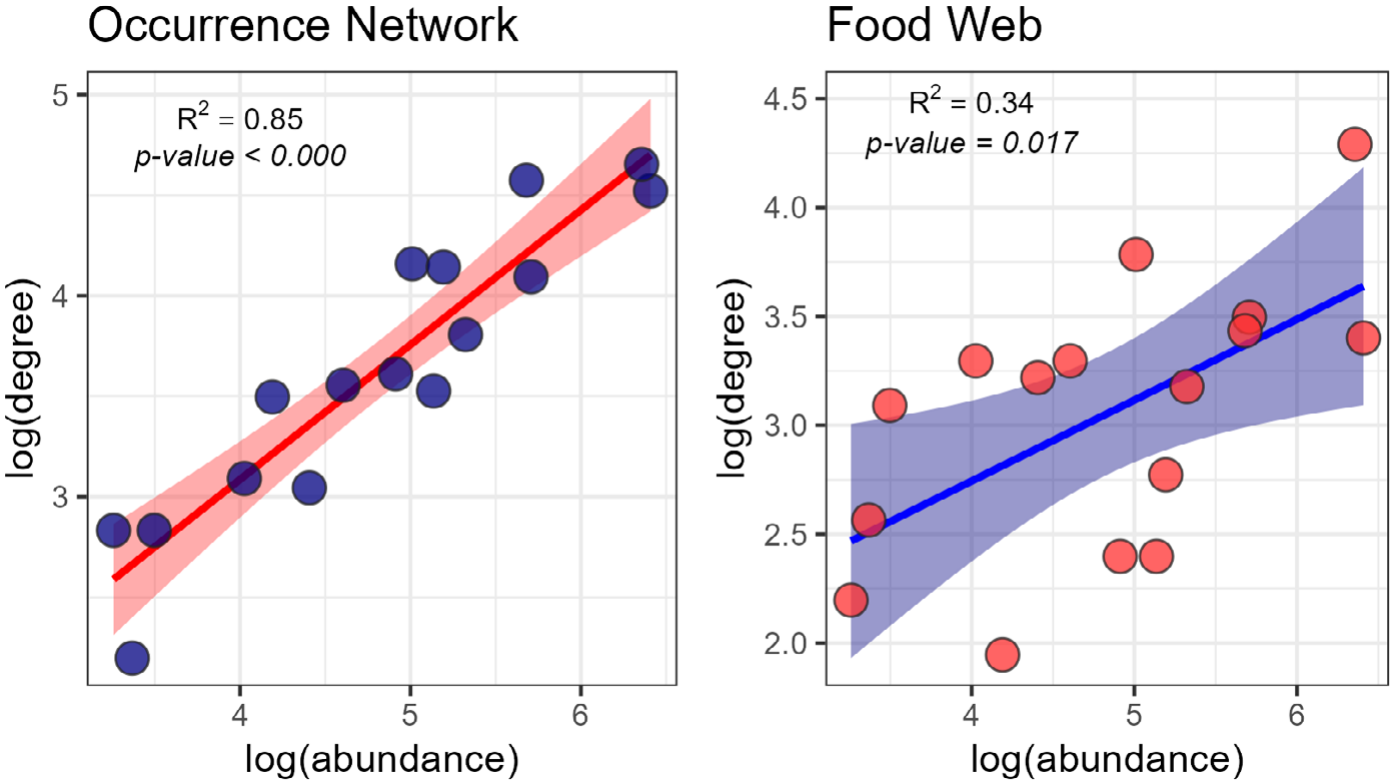
Relationship between the logarithm of predator abundance and degree in the occurrence networks (left in blue) and in the food web (right in red).

At the modular level, we detected contrasting patterns of intramodular nestedness. In the spatial network, three modules showed lower nestedness than expected by chance, whereas two exhibited higher nestedness (Figure 3a). In the food web, two modules showed lower nestedness and three displayed higher nestedness than expected (Figure 4a). Regarding functional dispersion (*Fdis*), different modules exhibited positive, negative and no deviation from random expectation, supporting processes of convergent selection, niche segregation and neutral processes, respectively (Figures 3b and 4b). Concerning the *CWM* of traits, the modules exhibited deviation of trait means from what was expected by chance (Figure 3c, lower panel, and Figure 4c, lower panel, Tables S5). For example, in the occurrence networks three modules grouped large predators (Body size >2 *z*, Figure 3c, Table S5), while Modules 2, 4, 5 and 6 were grouped small piscivorous (body size < −2 *z*, Figure 3c, Table S5). Other traits also deviated from expected by chance in occurrence networks, such as caudal fin aspect ratio, body elongation and caudal peduncle constriction; whereas in food webs, they were tooth shape, eye position and prey size in the food web. The values for intramodular nesting, functional diversity and CWMs for each module can be observed in Table S5 for occurrence networks and in Table S6 for food webs. Lastly, a significant positive relationship between species abundance and degree was observed in both the occurrence network (*R* ^2^ = 0.85, *F* = 90.31, *p* < 0.000) and the food web (*R* ^2^ = 0.31, *F* = 7.32, *p* < 0.017), indicating that as species become more abundant, they tend to be more generalists (Figure 5).

## DISCUSSION

The patterns we report for the Paraná River advance our understanding of how mechanisms promoting interactions and modular assembly shape both occurrence networks and food webs (see also Borzone Mas et al., 2022). First, niche‐based mechanisms were evidenced as determinants of trophic interactions and species occurrences. This involved a match between consumer traits and its resources and the environments used (e.g. environmental filters), and also, the morphological differentiation between species of the same spatial or trophic modules (e.g. stabilization of interaction by niche segregation). Second, neutral, trait‐independent mechanisms were also supported as a major determinant of these ecological networks. Species abundances were tightly associated with the number of connections in food webs and occurrence networks. It should be recognized that abundance could be determined by traits as body size and that the number of trophic connections and the range of spatial conditions inhabited by a species (Bascompte & Jordano, 2007). Not attempting to solve this chicken‐egg dilemma, our results support the idea that species abundance is an important piece for understanding both, the structure, and the interrelationship between ecological networks (Vázquez et al., 2005). Third, this study supports the idea that distinct mechanisms underlie modular organization, and that these mechanisms are not constant across modules. The marked variability in intramodular nestedness, functional dispersion and trait composition suggests that the assembly mechanism is contingent on each module, with different ecological processes potentially prevailing within the same network depending on the traits and species composition of each module. Taken together, these results highlight that the structure of occurrence networks and food webs in large river systems emerges from the joint and context‐dependent action of niche‐based and neutral processes, operating heterogeneously across modules rather than uniformly at the network level.

The observed association between resource use and predator traits highlights the importance of trait‐matching mechanisms in shaping modular network structure (Diniz & Aguiar, 2023; Vázquez et al., 2009). Predators of each module exploit specific habitats in the occurrence network and distinct prey types in the food web, grouping them by habitat use and consumption patterns (Rezende et al., 2009; Eskuche‐Keith et al., 2023, Figures 3c and 4c). Resources within a module are used by predators with similar traits, such as large body size, triangular teeth or low caudal fin aspect ratio (Eskuche‐Keith et al., 2023; Phillips et al., 2020). Body size can strongly shape module composition by influencing gape‐size limitation, energy need, movement capacity and prey diversity, driving the emergence of modular structure (Borzone Mas et al., 2022; Ortiz et al., 2023; Ortiz & Arim, 2016). In 10 of 13 modules (both in food webs and occurrence networks) predator body size showed a convergence in module composition (lower variation than expected by chance: SD in Figures 3c and 4c), further supporting its central role in resource partitioning and habitat use. Additionally, traits also significantly influenced predator–prey and predator–habitat interactions. In the occurrence network, traits like caudal peduncle constriction, caudal fin aspect ratio and body shape reflected a gradient in habitat use, from pelagic zones in large channels to macrophyte‐rich floodplain lakes (Bauer et al., 2021; Sutton et al., 2021). For example, high peduncle constriction and fusiform bodies, which are known to favour linear runs in open habitats, tend to overrepresent channel habitats in their modules, while low constriction and high fin aspect ratio, which are known to favour short bursts in structurally complex habitats, tend to overrepresent vegetated lakes in their modules (Borzone Mas et al., 2022; Breda et al., 2005; Webb, 1984). Concerning the food web, eye position distinguishes predators by their prey capture strategies: In general, predators that feed on gymnotids and characiforms have dorsal eyes that search for prey in the water column or near the surface; predators that capture benthic siluriforms have more ventral eyes since they feed close to the substrate; and serrasalmids, with large eyes and triangular teeth, are specialized for browsing fins and scales (Figure 4, Table S6). These traits appear to facilitate interactions and allow predators to overcome selective barriers by using resources within a module.

In addition to trait‐dependent mechanisms, species abundance also emerged as a key determinant of network structure and species interactions. Predator abundance was directly related to both the number of prey species consumed and the number of sites occupied (Figure 4). This abundance–degree relationship plays a critical role in network assembly through neutral mechanisms, where more abundant species are more likely to interact with a greater number of resources by chance. As a result, nested patterns can emerge and sometimes obscure modular organization (Canard et al., 2012; Poisot et al., 2015; Vázquez et al., 2009). In our study, the scaling between abundance and degree generated nested substructures within modules (three in the food web and two in the spatial network), but no evidence of a composite topology was found at the whole‐network level. Within these nested modules, once consumers overcome filtering effects and converge on module use, the most abundant predators tend to monopolize module's resources. This pattern suggests that modules may originate from evolutionary processes driving functional convergence, while the internal nestedness likely reflects recent ecological interactions among similar consumers (Felix et al., 2022; Mello et al., 2019; Pinheiro et al., 2022). For example, in the food web, the three modules with internal nested structure have the most abundant predators in the system: *Hoplias* spp., *S. maculatus* and juveniles of *S. brasiliensis* (Figure 4a). At the whole‐network level, the reduced degree of nestedness is driven by niche‐based mechanisms because of the high spatial heterogeneity and prey diversity in the Paraná River (Borzone Mas et al., 2025; Saigo et al., 2016; Scarabotti et al., 2017) can create forbidden interactions that limit the formation of nested networks and promote modular assembly.

In most modules, functional dispersion (FDis) was lower than expected by chance, indicating strong functional convergence. However, in some modules of the occurrence network, FDis was higher than expected. These latter modules were characterized by the selection of habitats, such as main river channels (modules of medium–large and medium–small main‐channel predators; Figure 3). Such habitats impose few constraints on predator–habitat use, resulting in a heterogeneous assemblage of species (Sutton et al., 2021). Main river channels provide stable hydrological conditions and continuous connectivity, allowing the colonization and persistence of a wide diversity of species (Mayora et al., 2020; Scarabotti et al., 2017). In contrast, lake habitats experience extreme temperature fluctuations, hypoxia and prolonged isolation, acting as strong ecological filters (Mayora et al., 2020; Scarabotti et al., 2011, 2017) that promote functional convergence processes in local assemblages. Regarding the food web, four out of six modules exhibited strong functional convergence, while only one showed a positive but non‐significant FDis value (Figure 4b). This latter module was composed mainly of invertebrate prey—small‐bodied organisms that impose minimal constraints related to prey size, defences or escape ability. Such prey serve as key food sources during early ontogenetic stages of piscivorous fish (Ortiz & Arim, 2016; Sánchez‐Hernández et al., 2019). Conversely, modules showing lower functional diversity suggest that groups of species specializing in particular prey types are morphologically differentiated from the rest of the assemblage. This pattern is consistent with an evolutionary ‘arms race’ dynamic, where trophic specialization emerges through predator–prey coevolution (Maliet et al., 2020). Module‐level specialization enhances system complementarity, while functional convergence may promote coexistence by reducing competitive displacement among species (Bauer et al., 2021; Borzone Mas et al., 2025; Chesson & Kuang, 2008).

In both types of networks, we observed convergent mechanisms and structural patterns. Niche‐based processes tend to compartmentalize interactions into modules, whereas abundance plays a predominant role in shaping degree distributions. Consequently, both networks exhibited a strong modular component and a lack of nestedness at the whole‐network level. One plausible explanation is that the balance between generalist–opportunist strategies and resource heterogeneity operates similarly in both networks, promoting convergent structures (Maliet et al., 2020). Supporting this view, we observed that niche‐ and abundance‐based mechanisms jointly shape how modules are assembled in relation to functional diversity, and how resource filters drive either convergence or divergence among modules. However, an alternative explanation is that the food web is not merely similar to, but partly a consequence of, the spatial network. Because predator distributions ultimately depend on the spatial distribution of prey and their ability to colonize habitats, trophic structure may partly emerge from the underlying spatial structure, despite or independent of the mechanisms that promote trophic interactions (Canavero et al., 2019; Westerbom et al., 2018). The modular structure of the spatial network restricts predator movement across the landscape, leading predators to primarily capture prey within their module of membership (Borzone Mas et al., 2022). This spatial compartmentalization could therefore shape the mechanisms underlying the structure of the food web (Warren, 1994). In this sense, the structure of the food web could emerge as a by‐product of the spatial configuration of the system.

Additionally, our results have some limitations that must be acknowledged. First, we only divided *S. brasiliensis* into ontogenetic stages due to its migratory nature and the abundance of data available. As shown here, juveniles and adults occupied different modules, suggesting that species roles and memberships may shift during ontogeny. Extending this approach to additional species could be important to better understand how inter‐ and intraspecific dynamics shape space use and prey utilization. Second, due to limited diet information for many taxa, our analyses were restricted to the 16 most abundant piscivorous species in an assemblage of 160 fish species recorded in the studied area. It remains an open question whether the same mechanisms identified here produce similar patterns in other guilds or at the community level. Finally, taxonomic resolution of prey items differed across groups, with invertebrates identified at the order level and fishes at the species level, which may have influenced module delineation and the interpretation of trophic patterns.

Our findings provide new insights into the structural patterns and mechanisms shaping food webs and occurrence networks, offering valuable implications for biodiversity conservation in the Middle Paraná River. First, the functional convergence of traits related to resource use enables predictions about the compartments that invaders are likely to occupy (David et al., 2017; Liew et al., 2016). By disentangling the influence of specific attributes on module assembly, we can infer the probable placement of invasive species within modules according to their traits. This information is critical for modelling invasion probabilities and forecasting competitive exclusions, particularly among species with similar body sizes but differing in life history strategies (Felix et al., 2022; Spaak & Schreiber, 2023). Second, our results indicate that forbidden interactions within food webs and occurrence networks might promote the modular organization of both networks. This underscores the importance of conserving prey and site heterogeneity to maintain the modularity of these networks, a property essential for preserving the ecosystem functioning of the Middle Paraná River (Borzone Mas et al., 2022, 2025; Pinheiro et al., 2019). Third, we observed that modular assembly processes vary across the landscape, with larger channels exhibiting greater functional diversity compared with floodplain water bodies (Figure 3c). Since the cohesion of both food web and occurrence networks heavily relies on the replacement of species with distinct functional attributes, preserving functional diversity emerges as a key conservation priority (Borzone Mas et al., 2022). In this sense, our results suggest that treating network modules as conservation units based on their assembly processes represents a promising strategy for maintaining essential functional diversity. This perspective offers a valuable framework for developing targeted conservation strategies (Borthagaray et al., 2018).

In this study, we provide empirical evidence of the structure of occurrence and food web networks, the assembly mechanisms governing modules, and the processes driving species interactions. Our findings reveal significant nesting at the modular level, but this does not scale to a composite topology at the network level. Within modules, resources aggregate on the basis of the type of environment in occurrence networks and prey type in food webs. Environmental and biological filters drive selection or competition mechanisms, shaping resource use in these modules. The use of these resources is both promoted and restricted by different traits, indicating that the adaptive value of predator traits is a key factor in network organization. Despite strong modularity and trait overlap, neutral dynamics are also a potential main determinant of network structures, suggesting the coexistence of deterministic and neutral processes. In particular, the adaptive value of traits promotes module formation through processes of selection or competition.

## AUTHOR CONTRIBUTIONS

Dalmiro Borzone Mas, Pablo Scarabotti and Matias Arim conceived the idea and conceptualization, and designed the methodology. Pablo Scarabotti, Patricio Alvarenga, Dalmiro Borzone Mas, Martin Vazquez and Pablo Vaschetto carried out the field sampling and the collection of individuals. Matias Arim, Pablo Scarabotti and Dalmiro Borzone Mas performed the statistical analyses. Dalmiro Borzone Mas, Matias Arim, Pablo Scarabotti, Martin Vazquez and Pablo Vaschetto wrote the manuscript. Dalmiro Borzone Mas, Patricio Alvarenga and Pablo Scarabotti did the graphic design. Dalmiro Borzone Mas performed the coding simulations. Pablo Scarabotti and Matias Arim raised the funds for this work. All authors contributed critically to the drafts and gave final approval for publication.

## CONFLICT OF INTEREST STATEMENT

All authors declare no conflict of interest.

## Supporting information


Material S1.

Material S2:

**Table S1:** Values for each morphological attribute of predators (see references in Material S1).

**Table S2:** Abundance [number of individuals], spatial degree [number of sites where it is present] and trophic degree [number of prey] for each species.

**Table S3:** Membership of each node to the module for the nodes of the occurrence network (sites and predators).

**Table S4:** Membership of each node to the module for the nodes of the food web (preys and predators).

**Table S5:** Functional diversity (Fdiv).

**Table S6:** Functional diversity (Fdiv).

## Data Availability

Data are available from the CONICET Digital Repository https://ri.conicet.gov.ar/handle/11336/278009 (Scarabotti et al. 2025).

## References

[jane70234-bib-0001] Almeida‐Neto, M. , Guimaraes, P. , Guimaraes, P., Jr. , Loyola, R. , & Ulrich, W. (2008). A consistent metric for nestedness analysis in ecological systems: Reconciling concept and measurement. Oikos, 117(8), 1227–1239.

[jane70234-bib-0002] Bartomeus, I. , Gravel, D. , Tylianakis, J. , Aizen, M. , Dickie, I. , & Bernard‐Verdier, M. (2016). A common framework for identifying linkage rules across different types of interactions. Functional Ecology, 30(12), 1894–1903.

[jane70234-bib-0003] Bascompte, J. , & Jordano, P. (2007). Plant‐animal mutualistic networks: The architecture of biodiversity. Annual Review of Ecology, Evolution, and Systematics, 38, 567–593.

[jane70234-bib-0004] Bauer, B. , Kleyer, M. , Albach, D. , Blasius, B. , Brose, U. , Ferreira‐Arruda, T. , Feudel, U. , Gerlach, G. , Hof, C. , Kreft, H. , Kuczynski, L. , Lõhmus, K. , Moorthi, S. , Scherber, C. , Scheu, S. , Zotz, G. , & Hillebrand, H. (2021). Functional trait dimensions of trophic metacommunities. Ecography, 44(10), 1486–1500.

[jane70234-bib-0005] Beckett, S. (2016). Improved community detection in weighted bipartite networks. Royal Society Open Science, 3(1), 140536. 26909160 10.1098/rsos.140536 PMC4736915

[jane70234-bib-0006] Borthagaray, A. , Arim, M. , & Marquet, P. (2014). Inferring species roles in metacommunity structure from species co‐occurrence networks. Proceedings of the Royal Society B: Biological Sciences, 281(1792), 20141425. 10.1098/rspb.2014.1425 PMC4150328 25143039

[jane70234-bib-0007] Borthagaray, A. , Soutullo, A. , Carranza, A. , & Arim, M. (2018). A modularity‐based approach for identifying biodiversity management units. Revista Chilena de Historia Natural, 91. https://www.scielo.cl/scielo.php?pid=S0716‐078X2018000100202&script=sci_arttext

[jane70234-bib-0008] Borzone Mas, D. , Scarabotti, P. , Alvarenga, P. , & Arim, M. (2022). Symmetries and asymmetries in the topological roles of piscivorous fishes between occurrence networks and food webs. Journal of Animal Ecology, 91, 2061–2073. 35869605 10.1111/1365-2656.13784

[jane70234-bib-0009] Borzone Mas, D. , Scarabotti, P. , Alvarenga, P. , Vaschetto, P. , & Arim, M. (2025). Food web structure mediates positive and negative effects of diversity on ecosystem functioning in a large floodplain river. The American Naturalist, 206(2), 115–129. 10.1086/735914 40720856

[jane70234-bib-0010] Breda, L. , de Fontes Oliveira, E. , & Goulart, E. (2005). Ecomorphology of fish locomotion with focus on Neotropical species. Acta Scientiarum. Biological Sciences, 27(4), 371–381.

[jane70234-bib-0011] Canard, E. , Mouquet, N. , Marescot, L. , Gaston, K. , Gravel, D. , & Mouillot, D. (2012). Emergence of structural patterns in neutral trophic networks. 10.1371/journal.pone.0038295 PMC3416803 22899987

[jane70234-bib-0012] Canavero, A. , Arim, M. , Perez, F. , Jaksic, F. , & Marquet, P. (2019). Phenological modularity in amphibian calling behaviour: Geographic trends and local determinants. Austral Ecology, 44(8), 1451–1462.

[jane70234-bib-0013] Chesson, P. , & Kuang, J. (2008). The interaction between predation and competition. Nature, 456(7219), 235–238. 19005554 10.1038/nature07248

[jane70234-bib-1007] Claverie, T. , & Wainwright, P. C. (2014). A morphospace for reef fishes: Elongation is the dominant axis of body shape evolution. PLoS One, 9(11), e112732. 25409027 10.1371/journal.pone.0112732 PMC4237352

[jane70234-bib-1008] CONICET (Consejo Nacional de Investigaciones Científicas y Técnicas) . (2005). Marco Ético de Referencia para las Investigaciones Biomédicas en Animales de laboratorio, de granja y obtenidos de la naturaleza, Buenos Aires, Argentina. CONICET.

[jane70234-bib-0014] David, P. , Thebault, E. , Anneville, O. , Duyck, P. , Chapuis, E. , & Loeuille, N. (2017). Impacts of invasive species on food webs: A review of empirical data. Advances in Ecological Research, 56, 1–60.

[jane70234-bib-0015] Dehling, D. , Lai, H. , & Stouffer, D. (2025). Eltonian niche modelling: Applying joint hierarchical niche models to ecological networks. Ecology Letters, 28, e70120. 40439603 10.1111/ele.70120 PMC12121635

[jane70234-bib-0016] Delmas, E. , Besson, M. , Brice, M. , Burkle, L. , Dalla Riva, G. V. , Fortin, M.‐J. , Gravel, D. , Guimarães, P. R., Jr. , Hembry, D. H. , Newman, E. A. , Olesen, J. M. , Pires, M. M. , Yeakel, J. D. , & Poisot, T. (2019). Analysing ecological networks of species interactions. Biological Reviews, 94(1), 16–36. 29923657 10.1111/brv.12433

[jane70234-bib-0017] Diniz, U. , & Aguiar, L. (2023). The interplay between spatiotemporal overlap and morphology as determinants of microstructure suggests no ‘perfect fit’ in a bat‐flower network. Scientific Reports, 13(1), 2737. 36792891 10.1038/s41598-023-29965-3 PMC9932087

[jane70234-bib-0018] Dormann, C. , Gruber, B. , & Fründ, J. (2008). Introducing the bipartite package: Analysing ecological networks. Interaction, 8/2, 8–11.

[jane70234-bib-0019] Doulcier, G. , & Stouffer, D. (2015). Rnetcarto: Fast network modularity and roles computation by simulated annealing R package.

[jane70234-bib-0020] Eskuche‐Keith, P. , Hill, S. , Hollyman, P. , Taylor, M. , & O'Gorman, E. (2023). Trophic structuring of modularity alters energy flow through marine food webs. Frontiers in Marine Science, 9, 1046150.

[jane70234-bib-0021] Felix, G. , Pinheiro, R. , Poulin, R. , Krasnov, B. , & Mello, M. (2022). The compound topology of host–parasite networks is explained by the integrative hypothesis of specialization. Oikos, 2022(1). https://nsojournals.onlinelibrary.wiley.com/doi/epdf/10.1111/oik.08462

[jane70234-bib-0022] Fortuna, M. , Popa‐Lisseanu, A. , Ibáñez, C. , & Bascompte, J. (2009). The roosting spatial network of a bird‐predator bat. Ecology, 90(4), 934–944. 19449689 10.1890/08-0174.1

[jane70234-bib-0023] Guimaraes, P., Jr. (2020). The structure of ecological networks across levels of organization. Annual Review of Ecology, Evolution, and Systematics, 51, 433–460.

[jane70234-bib-0024] Guimera, R. , & Amaral, L. (2005). Cartography of complex networks: Modules and universal roles. Journal of Statistical Mechanics: Theory and Experiment, 2005(2), P02001. 10.1088/1742-5468/2005/02/P02001 PMC2151742 18159217

[jane70234-bib-0025] Hammerschlag, N. , Schmitz, O. , Flecker, A. , Lafferty, K. , Schmitz, O. J. , Flecker, A. S. , Lafferty, K. D. , Sih, A. , Atwood, T. B. , Gallagher, A. J. , Irschick, D. J. , Skubel, R. , Cooke, S. J. , & Cooke, S. (2019). Ecosystem function and services of aquatic predators in the Anthropocene. Trends in Ecology & Evolution, 34(4), 369–383. 30857757 10.1016/j.tree.2019.01.005

[jane70234-bib-1005] HilleRisLambers, J. , Adler, P. B. , Harpole, W. S. , Levine, J. M. , & Mayfield, M. M. (2012). Rethinking community assembly through the lens of coexistence theory. Annual Review of Ecology, Evolution, and Systematics, 43(1), 227–248.

[jane70234-bib-0026] Izquierdo‐Palma, J. , del Coro Arizmendi, M. , Lara, C. , & Ornelas, J. (2021). Forbidden links, trait matching and modularity in plant‐hummingbird networks: Are specialized modules characterized by higher phenotypic floral integration? PeerJ, 9, e10974. 33854834 10.7717/peerj.10974 PMC7955668

[jane70234-bib-0027] Laliberté, E. , & Legendre, P. (2010). A distance‐based framework for measuring functional diversity from multiple traits. Ecology, 91(1), 299–305. 20380219 10.1890/08-2244.1

[jane70234-bib-0028] Legras, G. , Loiseau, N. , Gaertner, J. , Poggiale, J. , Ienco, D. , Mazouni, N. , & Mérigot, B. (2019). Assessment of congruence between co‐occurrence and functional networks: A new framework for revealing community assembly rules. Scientific Reports, 9(1), 19996. 31882755 10.1038/s41598-019-56515-7 PMC6934466

[jane70234-bib-1001] Lewinsohn, T. M. , Inácio Prado, P. , Jordano, P. , Bascompte, J. , & Olesen, J. M. (2006). Structure in plant–animal interaction assemblages. Oikos, 113(1), 174–184.

[jane70234-bib-0029] Liew, J. , Carrasco, L. , Tan, H. , & Yeo, D. (2016). Native richness and species level trophic traits predict establishment of alien freshwater fishes. Biological Invasions, 18, 3495–3512.

[jane70234-bib-0030] Maliet, O. , Loeuille, N. , & Morlon, H. (2020). An individual‐based model for the eco‐evolutionary emergence of bipartite interaction networks. Ecology Letters, 23(11), 1623–1634. 32885919 10.1111/ele.13592

[jane70234-bib-0031] Mariani, M. , Ren, Z. , Bascompte, J. , & Tessone, C. (2019). Nestedness in complex networks: Observation, emergence, and implications. Physics Reports, 813, 1–90.

[jane70234-bib-1003] Marjakangas, E. L. , Muñoz, G. , Turney, S. , Albrecht, J. , Neuschulz, E. L. , Schleuning, M. , & Lessard, J. P. (2022). Trait‐based inference of ecological network assembly: A conceptual framework and methodological toolbox. Ecological Monographs, 92(2), e1502.

[jane70234-bib-0032] Mayora, G. , Scarabotti, P. , Schneider, B. , Alvarenga, P. , & Marchese, M. (2020). Multiscale environmental heterogeneity in a large river‐floodplain system. Journal of South American Earth Sciences, 100, 102546.

[jane70234-bib-1010] Mello, M. A. , Felix, G. M. , Pinheiro, R. B. , Muylaert, R. L. , Geiselman, C. , Santana, S. E. , Tschapka, M. , Lotfi, N. , Rodrigues, F. A. , & Stevens, R. D. (2019). Insights into the assembly rules of a continent‐wide multilayer network. Nature Ecology & Evolution, 3(11), 1525–1532. 31611677 10.1038/s41559-019-1002-3

[jane70234-bib-0033] Mintrone, C. , Rindi, L. , Bertocci, I. , Maggi, E. , & Benedetti‐Cecchi, L. (2025). Modularity buffers the spread of spatial perturbations in macroalgal networks. Current Biology, 35(1), 154–162. 39706172 10.1016/j.cub.2024.11.038

[jane70234-bib-0034] Mouillot, D. , Dumay, O. , & Tomasini, J. (2007). Limiting similarity, niche filtering and functional diversity in coastal lagoon fish communities. Estuarine, Coastal and Shelf Science, 71(3–4), 443–456.

[jane70234-bib-0035] Newman, M. E. (2006). Modularity and community structure in networks. Proceedings of the National Academy of Sciences of the United States of America, 103, 8577–8582. 16723398 10.1073/pnas.0601602103 PMC1482622

[jane70234-bib-0036] Nie, S. , Zheng, J. , Luo, M. , Loreau, M. , Gravel, D. , & Wang, S. (2023). Will a large complex system be productive? Ecology Letters, 26, 1325–1335. 37190868 10.1111/ele.14242

[jane70234-bib-0037] Olesen, J. , Bascompte, J. , Dupont, Y. , & Jordano, P. (2007). The modularity of pollination networks. Proceedings of the National Academy of Sciences of the United States of America, 104(50), 19891–19896. 18056808 10.1073/pnas.0706375104 PMC2148393

[jane70234-bib-0038] Omidipour, R. , Tahmasebi, P. , Faizabadi, M. , Faramarzi, M. , & Ebrahimi, A. (2021). Does β diversity predict ecosystem productivity better than species diversity? Ecological Indicators, 122, 107212.

[jane70234-bib-0039] Ortiz, E. , & Arim, M. (2016). Hypotheses and trends on how body size affects trophic interactions in a guild of South American killifishes. Austral Ecology, 41(8), 976–982.

[jane70234-bib-0040] Ortiz, E. , Ramos‐Jiliberto, R. , & Arim, M. (2023). Prey selection along a predators' body size gradient evidences the role of different trait‐based mechanisms in food web organization. PLoS One, 18(10), e0292374. 37797081 10.1371/journal.pone.0292374 PMC10553361

[jane70234-bib-0041] Phillips, R. , Peakall, R. , van der Niet, T. , & Johnson, S. (2020). Niche perspectives on plant–pollinator interactions. Trends in Plant Science, 25(8), 779–793. 32386827 10.1016/j.tplants.2020.03.009

[jane70234-bib-1004] Pinheiro, R. B. , Felix, G. M. , Chaves, A. V. , Lacorte, G. A. , Santos, F. R. , Braga, E. M. , & Mello, M. A. (2016). Trade‐offs and resource breadth processes as drivers of performance and specificity in a host–parasite system: A new integrative hypothesis. International Journal for Parasitology, 46(2), 115–121. 26552015 10.1016/j.ijpara.2015.10.002

[jane70234-bib-0042] Pinheiro, R. , Felix, G. , Dormann, C. , & Mello, M. (2019). A new model explaining the origin of different topologies in interaction networks. Ecology, 100(9), e02796. 31232470 10.1002/ecy.2796

[jane70234-bib-0043] Pinheiro, R. , Felix, G. , & Lewinsohn, T. (2022). Hierarchical compound topology uncovers complex structure of species interaction networks. Journal of Animal Ecology, 91(11), 2248–2260. 36054553 10.1111/1365-2656.13806

[jane70234-bib-1009] Poisot, T. , Stouffer, D. B. , & Gravel, D. (2015). Beyond species: Why ecological interaction networks vary through space and time. Oikos, 124(3), 243–251.

[jane70234-bib-0044] R Core Team . (2022). R: A language and environment for statistical computing. R Foundation for Statistical Computing. https://www.R‐project.org/

[jane70234-bib-0045] Rezende, E. , Albert, E. , Fortuna, M. , & Bascompte, J. (2009). Compartments in a marine food web associated with phylogeny, body mass, and habitat structure. Ecology Letters, 12(8), 779–788. 19490028 10.1111/j.1461-0248.2009.01327.x

[jane70234-bib-1002] Rodriguez, I. D. , Marina, T. I. , Schloss, I. R. , & Saravia, L. A. (2022). Marine food webs are more complex but less stable in sub‐Antarctic (Beagle Channel, Argentina) than in Antarctic (Potter Cove, Antarctic Peninsula) regions. Marine Environmental Research, 174, 105561. 35026725 10.1016/j.marenvres.2022.105561

[jane70234-bib-0046] Rossi, L. , Cordiviola, E. , & Parma, M. (2007). Fishes. In M. H. Iriondo , J. C. Paggi , & M. J. Parma (Eds.), The middle Paraná River: Limnology of a subtropical wetland (pp. 305–326). Springer‐Verlag.

[jane70234-bib-0047] Saigo, M. , Marchese, M. , & Wantzen, K. (2016). A closer look at the main actors of Neotropical floodplain food webs: Functional classification and niche overlap of dominant benthic invertebrates in a floodplain lake of Paraná River. Iheringia. Série Zoologia, 106. https://www.bvs‐vet.org.br/vetindex/periodicos/iheringia‐serie‐zoologia/106‐(2016)/a‐closer‐look‐at‐the‐main‐actors‐of‐neotropical‐floodplain‐food‐webs‐f/

[jane70234-bib-0048] Sánchez‐Hernández, J. , Nunn, A. , Adams, C. , & Amundsen, P. (2019). Causes and consequences of ontogenetic dietary shifts: A global synthesis using fish models. Biological Reviews, 94(2), 539–554. 30251433 10.1111/brv.12468

[jane70234-bib-0049] Scarabotti, P. , Demonte, L. , & Pouilly, M. (2017). Climatic seasonality, hydrological variability, and geomorphology shape fish assemblage structure in a subtropical floodplain. Freshwater Science, 36(3), 653–668.

[jane70234-bib-0050] Scarabotti, P. , Lopez, J. , & Pouilly, M. (2011). Flood pulse and the dynamics of fish assemblage structure from Neotropical floodplain lakes. Ecology of Freshwater Fish, 20(4), 605–618.

[jane70234-bib-0051] Scarabotti, P. A. , Borzone Mas, D. , & Alvarenga, P. F. (2025). Occurrence and feeding interactions data on 16 piscivororus fish species of the middle Paraná River [Dataset]. Version of December 20. Consejo Nacional de Investigaciones Científicas y Técnicas.

[jane70234-bib-0052] Spaak, J. , & Schreiber, S. (2023). Building modern coexistence theory from the ground up: The role of community assembly. Ecology Letters, 26(11), 1840–1861. 37747362 10.1111/ele.14302

[jane70234-bib-0053] Sutton, L. , Mueter, F. , Bluhm, B. , & Iken, K. (2021). Environmental filtering influences functional community assembly of epibenthic communities. Frontiers in Marine Science, 8, 736917.

[jane70234-bib-0054] Thébault, E. , & Fontaine, C. (2010). Stability of ecological communities and the architecture of mutualistic and trophic networks. Science, 329(5993), 853–856. 20705861 10.1126/science.1188321

[jane70234-bib-0055] Trøjelsgaard, K. , & Olesen, J. (2013). Macroecology of pollination networks. Global Ecology and Biogeography, 22(2), 149–162.

[jane70234-bib-0056] Vázquez, D. (2005). Degree distribution in plant–animal mutualistic networks: Forbidden links or random interactions? Oikos, 108, 421–426.

[jane70234-bib-0057] Vázquez, D. , Blüthgen, N. , Cagnolo, L. , & Chacoff, N. (2009). Uniting pattern and process in plant–animal mutualistic networks: A review. Annals of Botany, 103(9), 1445–1457. 19304996 10.1093/aob/mcp057 PMC2701748

[jane70234-bib-0058] Vázquez, D. , Melián, C. J. , Williams, N. M. , Blüthgen, N. , Krasnov, B. R. , & Poulin, R. (2007). Species abundance and asymmetric interaction strength in ecological networks. Oikos, 116(7), 1120–1127.

[jane70234-bib-0059] Vázquez, D. , Poulin, R. , Krasnov, B. , & Poulin, R. (2005). Species abundance and the distribution of specialization in host–parasite interaction networks. Journal of Animal Ecology, 74, 946–955.

[jane70234-bib-0060] Villéger, S. , Novack‐Gottshall, P. , & Mouillot, D. (2011). The multidimensionality of the niche reveals functional diversity changes in benthic marine biotas across geological time. Ecology Letters, 14(6), 561–568. 21481126 10.1111/j.1461-0248.2011.01618.x

[jane70234-bib-0061] Vizentin‐Bugoni, J. , Maruyama, P. , & Sazima, M. (2014). Processes entangling interactions in communities: Forbidden links are more important than abundance in a hummingbird–plant network. Proceedings of the Royal Society B: Biological Sciences, 281(1780), 20132397. 10.1098/rspb.2013.2397 PMC4027382 24552835

[jane70234-bib-0062] Warren, P. (1994). Making connections in food webs. Trends in Ecology & Evolution, 9(4), 136–141. 21236797 10.1016/0169-5347(94)90178-3

[jane70234-bib-0063] Webb, P. (1984). Form and function in fish swimming. Scientific American, 251(1), 72–83.

[jane70234-bib-0064] Westerbom, M. , Lappalainen, A. , Mustonen, O. , & Norkko, A. (2018). Trophic overlap between expanding and contracting fish predators in a range margin undergoing change. Scientific Reports, 8(1), 7895. 29785034 10.1038/s41598-018-25745-6 PMC5962582

[jane70234-bib-1006] Winemiller, K. O. (1991). Ecomorphological diversification in lowland freshwater fish assemblages from five biotic regions. Ecological Monographs, 61(4), 343–365.

