# Supplementary material for: On traits matching and the modular organization of food web and occurrence networks
Source: J Anim Ecol. 2026 Mar 16;95(5):837–50. doi: 10.1111/1365-2656.70234 (PMC13145326; doi:10.1111/1365-2656.70234)
Supplement: Supplementary file 1 — Material S1. [file JANE-95-837-s004.docx]

Supplemental material 1:


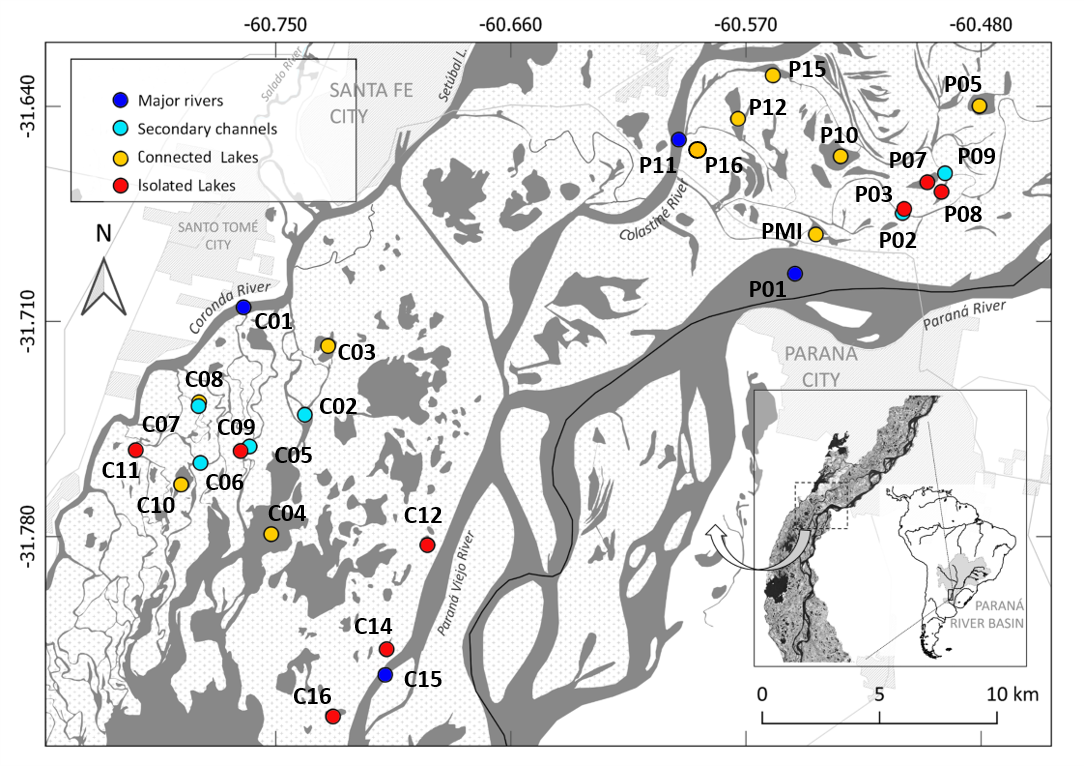


Study area showing the spatial distribution of sampling sites. The color of each site indicates its habitat type. Below, we present the sampling date (Date), survey identifier (Camp), habitat type (HT), and total fish abundance recorded at each site. We distinguished between two types of surveys: spatial (labeled “M”) and temporal (labeled “T”). Spatial surveys aimed to capture the distribution of fish assemblages across the floodplain by sampling a large number of sites on four occasions. Temporal surveys focused on capturing community dynamics over time, by repeatedly sampling four representative water bodies every two months over approximately two years. Because in some cases sites were inaccessible (e.g., due to dry conditions) or no predators were captured, the total number of analyzed communities did not match the expected number of samples.

| site | camp | HT | abundance | date |
| --- | --- | --- | --- | --- |
| C01 | M1 | MR | 41 | 11/19/2013 |
| C01 | M2 | MR | 25 | 03/24/2014 |
| C01 | M3 | MR | 7 | 08/31/2015 |
| C01 | M4 | MR | 8 | 03/31/2016 |
| C02 | M1 | SC | 10 | 11/19/2013 |
| C02 | M2 | SC | 3 | 03/24/2014 |
| C02 | M3 | SC | 17 | 08/31/2015 |
| C02 | M4 | SC | 9 | 03/31/2016 |
| C03 | M1 | CL | 47 | 11/19/2013 |
| C03 | M3 | CL | 25 | 08/31/2015 |
| C03 | M4 | CL | 11 | 03/31/2016 |
| C04 | M1 | CL | 24 | 11/19/2013 |
| C04 | M2 | CL | 6 | 03/24/2014 |
| C04 | M4 | CL | 3 | 03/31/2016 |
| C05 | M1 | SC | 4 | 11/19/2013 |
| C05 | M2 | SC | 4 | 03/24/2014 |
| C05 | M3 | SC | 8 | 08/31/2015 |
| C05 | M4 | SC | 5 | 03/31/2016 |
| C06 | M1 | IL | 19 | 11/19/2013 |
| C06 | M2 | IL | 6 | 03/24/2014 |
| C06 | M3 | IL | 14 | 08/31/2015 |
| C06 | M4 | IL | 10 | 03/31/2016 |
| C07 | M1 | SC | 13 | 11/19/2013 |
| C07 | M2 | SC | 19 | 03/24/2014 |
| C07 | M3 | SC | 5 | 08/31/2015 |
| C07 | M4 | SC | 39 | 03/31/2016 |
| C07 | T01 | SC | 4 | 11/04/2015 |
| C07 | T02 | SC | 8 | 12/15/2015 |
| C07 | T04 | SC | 23 | 05/11/2016 |
| C07 | T05 | SC | 40 | 07/05/2016 |
| C07 | T06 | SC | 46 | 08/17/2016 |
| C07 | T07 | SC | 22 | 10/11/2016 |
| C07 | T08 | SC | 24 | 11/22/2016 |
| C07 | T09 | SC | 33 | 12/28/2016 |
| C07 | T10 | SC | 10 | 03/14/2017 |
| C07 | T11 | SC | 3 | 05/23/2017 |
| C07 | T12 | SC | 27 | 08/07/2017 |
| C07 | T13 | SC | 5 | 10/24/2017 |
| C07 | T14 | SC | 11 | 12/22/2017 |
| C08 | M1 | CL | 12 | 11/19/2013 |
| C08 | M2 | CL | 16 | 03/24/2014 |
| C08 | M3 | CL | 36 | 08/31/2015 |
| C08 | M4 | CL | 3 | 03/31/2016 |
| C09 | M1 | SC | 12 | 11/19/2013 |
| C09 | M2 | SC | 4 | 03/24/2014 |
| C09 | M3 | SC | 11 | 08/31/2015 |
| C09 | M4 | SC | 14 | 03/31/2016 |
| C10 | M1 | CL | 8 | 11/19/2013 |
| C10 | M2 | CL | 5 | 03/24/2014 |
| C10 | M3 | CL | 6 | 08/31/2015 |
| C10 | M4 | CL | 15 | 03/31/2016 |
| C11 | M1 | IL | 15 | 11/19/2013 |
| C11 | M2 | IL | 37 | 03/24/2014 |
| C11 | M3 | IL | 15 | 08/31/2015 |
| C11 | M4 | IL | 37 | 03/31/2016 |
| C11 | T01 | IL | 11 | 11/04/2015 |
| C11 | T02 | IL | 41 | 12/15/2015 |
| C11 | T04 | IL | 7 | 05/11/2016 |
| C11 | T05 | IL | 56 | 07/05/2016 |
| C11 | T06 | IL | 7 | 08/17/2016 |
| C11 | T07 | IL | 20 | 10/11/2016 |
| C11 | T08 | IL | 23 | 11/22/2016 |
| C11 | T09 | IL | 54 | 12/28/2016 |
| C11 | T10 | IL | 21 | 03/14/2017 |
| C11 | T11 | IL | 4 | 05/23/2017 |
| C11 | T12 | IL | 8 | 08/07/2017 |
| C11 | T13 | IL | 12 | 10/24/2017 |
| C11 | T14 | IL | 30 | 12/22/2017 |
| C12 | M1 | IL | 2 | 11/19/2013 |
| C12 | M2 | IL | 13 | 03/24/2014 |
| C12 | M3 | IL | 1 | 08/31/2015 |
| C12 | M4 | IL | 9 | 03/31/2016 |
| C14 | M1 | IL | 8 | 11/19/2013 |
| C14 | M2 | IL | 14 | 03/24/2014 |
| C14 | M3 | IL | 19 | 08/31/2015 |
| C14 | M4 | IL | 7 | 03/31/2016 |
| C15 | M1 | MR | 32 | 11/19/2013 |
| C15 | M2 | MR | 17 | 03/24/2014 |
| C15 | M3 | MR | 39 | 08/31/2015 |
| C15 | M4 | MR | 18 | 03/31/2016 |
| C16 | M1 | IL | 3 | 11/19/2013 |
| C16 | M4 | IL | 4 | 03/31/2016 |
| P01 | M1 | MR | 17 | 11/19/2013 |
| P01 | M4 | MR | 2 | 03/31/2016 |
| P02 | M1 | SC | 19 | 11/19/2013 |
| P02 | M2 | SC | 31 | 03/24/2014 |
| P02 | M3 | SC | 22 | 08/31/2015 |
| P02 | M4 | SC | 11 | 03/31/2016 |
| P03 | M2 | IL | 1 | 03/24/2014 |
| P05 | M1 | CL | 16 | 11/19/2013 |
| P05 | M2 | CL | 72 | 03/24/2014 |
| P05 | M3 | CL | 19 | 08/31/2015 |
| P05 | M4 | CL | 1 | 03/31/2016 |
| P07 | M1 | IL | 15 | 11/19/2013 |
| P07 | M3 | IL | 29 | 08/31/2015 |
| P07 | M4 | IL | 5 | 03/31/2016 |
| P08 | M1 | IL | 5 | 11/19/2013 |
| P08 | M3 | IL | 16 | 08/31/2015 |
| P08 | M4 | IL | 13 | 03/31/2016 |
| P09 | M1 | SC | 3 | 11/19/2013 |
| P09 | M2 | SC | 15 | 03/24/2014 |
| P09 | M3 | SC | 16 | 08/31/2015 |
| P09 | M4 | SC | 4 | 03/31/2016 |
| P10 | M3 | CL | 13 | 08/31/2015 |
| P10 | M4 | CL | 8 | 03/31/2016 |
| P11 | M1 | MR | 37 | 11/19/2013 |
| P11 | M2 | MR | 22 | 03/24/2014 |
| P11 | M3 | MR | 4 | 08/31/2015 |
| P11 | M4 | MR | 9 | 03/31/2016 |
| P11 | T01 | MR | 40 | 11/04/2015 |
| P11 | T02 | MR | 28 | 12/15/2015 |
| P11 | T03 | MR | 12 | 01/07/2016 |
| P11 | T04 | MR | 110 | 05/11/2016 |
| P11 | T05 | MR | 64 | 07/05/2016 |
| P11 | T06 | MR | 66 | 08/17/2016 |
| P11 | T07 | MR | 10 | 10/11/2016 |
| P11 | T08 | MR | 43 | 11/22/2016 |
| P11 | T09 | MR | 43 | 12/28/2016 |
| P11 | T10 | MR | 25 | 03/14/2017 |
| P11 | T11 | MR | 1 | 05/23/2017 |
| P11 | T12 | MR | 24 | 08/07/2017 |
| P11 | T13 | MR | 33 | 10/24/2017 |
| P11 | T14 | MR | 40 | 12/22/2017 |
| P12 | M1 | CL | 36 | 11/19/2013 |
| P12 | M2 | CL | 34 | 03/24/2014 |
| P12 | M3 | CL | 15 | 08/31/2015 |
| P12 | M4 | CL | 3 | 03/31/2016 |
| P15 | M1 | CL | 8 | 11/19/2013 |
| P15 | M2 | CL | 27 | 03/24/2014 |
| P15 | M3 | CL | 44 | 08/31/2015 |
| P15 | M4 | CL | 8 | 03/31/2016 |
| P16 | T01 | CL | 48 | 11/04/2015 |
| P16 | T02 | CL | 7 | 12/15/2015 |
| P16 | T03 | CL | 37 | 01/07/2016 |
| P16 | T04 | CL | 7 | 05/11/2016 |
| P16 | T05 | CL | 25 | 07/05/2016 |
| P16 | T06 | CL | 38 | 08/17/2016 |
| P16 | T07 | CL | 31 | 10/11/2016 |
| P16 | T08 | CL | 33 | 11/22/2016 |
| P16 | T09 | CL | 66 | 12/28/2016 |
| P16 | T10 | CL | 39 | 03/14/2017 |
| P16 | T11 | CL | 2 | 05/23/2017 |
| P16 | T12 | CL | 9 | 08/07/2017 |
| P16 | T13 | CL | 69 | 10/24/2017 |
| P16 | T14 | CL | 28 | 12/22/2017 |
| PMi | M1 | CL | 22 | 11/19/2013 |
| PMi | M2 | CL | 20 | 03/24/2014 |
| PMi | M3 | CL | 17 | 08/31/2015 |
| PMi | M4 | CL | 9 | 03/31/2016 |
