# Supplementary material for: On traits matching and the modular organization of food web and occurrence networks
Source: J Anim Ecol. 2026 Mar 16;95(5):837–50. doi: 10.1111/1365-2656.70234 (PMC13145326; doi:10.1111/1365-2656.70234)
Supplement: Supplementary file 2 — Material S2: [file JANE-95-837-s001.doc]

**Supplemental material 1**

List of the 12 functional traits used. The codes for the morphological measurements can be seen in the figure on the next page. The functional trait index above indicates the reference (adapted from supplemental material of Villéger et al. 2012).

| **Functional trait** | **Formula** | **Ecological meaning** |
| --- | --- | --- |
| Body elongation^1^ | 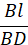 | Maniobravilidad y capacidad de natación |
| Oral gape size^2^ | $\frac{Mw\times Md}{Bw\times Bd}$ | Nature/Size of food items captured |
| Oral gape shape^2^ | $\frac{Md}{Mw}$ | Method to capture food items |
| Digestive length^2^ | $\frac{Gl}{Bl}$ | Processing of energy poor resources such as vegetation and detritus |
| Eyes size^2^ | $\frac{Ed}{Hd}$ | Prey detection |
| Eyes position^2^ | $\frac{Eh}{Hd}$ | Vertical position in the water column |
| Body transversal shape | $\frac{Bd}{Bw}$ | Vertical position in the water column and hydrodynamism |
| Caudal peduncle throttling^2^ | $\frac{CFd}{CPd}$ | Caudal propulsion efficiency through reduction of drag |
| Caudal aspecto ratio^2^ | $\frac{CFd^{2}}{CFs}$ | Caudal fin use for propulsion and/or direction |
| Pigmentation code^3^ | categorical | 0 = transparent, 1 = reflective silver, 2 = silver with vertical spots, 3 = light colors with backshading, 4 = side or vertical bars with backshading, 5 = densely spotted, and 6 = black |
| Teeth shape^3^ | categorical | 0 = Absent, 1 = Scraping unicuspids, 2 = Crushing multicuspids, 3 = Short conical, 4 = Long conical and 5 = Triangular |

| **Figure** | **Trait** |
| --- | --- |
| **Ed** | Eye diameter |
| **Hd** | Head height |
| **Bl** | Standard length |
| **Cpd** | Caudal peduncle height |
| **CFs** | Caudal fin área |
| **CFd** | Caudal fin height |
| **Bd** | Body height |
| **Bw** | Maximum width |
| **Md** | Mouth height |
| **Mw** | Mouth width |


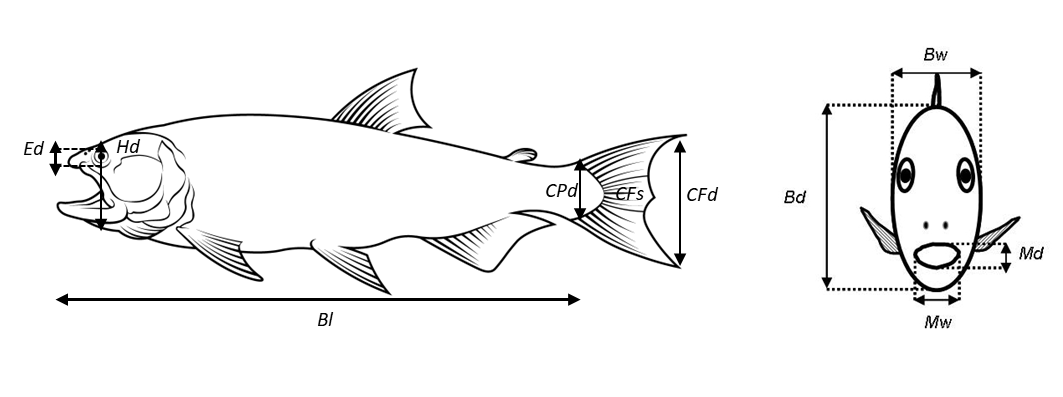


**References**

1. Claverie, T., & Wainwright, P. C. (2014). A morphospace for reef fishes: elongation is the dominant axis of body shape evolution. *PloS one*, *9*(11), e112732.

2. Villéger, S., Miranda, J. R., Hernandez, D. F., & Mouillot, D. (2012). Low functional β-diversity despite high taxonomic β-diversity among tropical estuarine fish communities. *PloS one*, *7*(7), e40679.

3. Winemiller, K. O. (1991). Ecomorphological diversification in lowland freshwater fish assemblages from five biotic regions. *Ecological Monographs*, *61*(4), 343-365.
